# Systematic profiling of cellular phenotypes with spotted cell microarrays reveals mating-pheromone response genes

**DOI:** 10.1186/gb-2006-7-1-r6

**Published:** 2006-01-31

**Authors:** Rammohan Narayanaswamy, Wei Niu, Alexander D Scouras, G Traver Hart, Jonathan Davies, Andrew D Ellington, Vishwanath R Iyer, Edward M Marcotte

**Affiliations:** 1 Center for Systems and Synthetic Biology, Institute for Cellular and Molecular Biology, 2500 Speedway, University of Texas, Austin, TX 78712, USA

## Abstract

We have developed spotted cell microarrays for measuring cellular phenotypes on a large scale. Collections of cells are printed, stained for subcellular features, then imaged via automated, high-throughput microscopy, allowing systematic phenotypic characterization. We used this technology to identify genes involved in the response of yeast to mating pheromone. Besides morphology assays, cell microarrays should be valuable for high-throughput *in situ *hybridization and immunoassays, enabling new classes of genetic assays based on cell imaging.

## Abstract 2

Spotted cell microarrays were developed for measuring cellular phenotypes on a large scale and used to identify genes involved in the response of yeast to mating pheromone.

## Background

A major goal in functional genomics, proteomics, and systems biology is to define the biological functions of the genes encoded in each genome and to reconstruct the network of functional interactions that underlies normal and altered cellular and organismal biology [1]. DNA microarrays, mass spectrometry, and protein-interaction screens have been powerful tools in this regard [2,3], but it is important to employ diverse technologies addressing independent aspects of gene function in order to generate complementary datasets [4]. In particular, spatial, temporal and phenotypic data provide important clues for understanding genetic circuitry.

In this paper, we describe a technology for measuring cell morphology and subcellular localization phenotypes, applied to a model system in which yeast change morphology in response to mating pheromone [5,6]. Wild-type haploid yeast cells, on detecting pheromone of the opposite mating type via a cell surface receptor, heterotrimeric G protein, and mitogen-activated protein (MAP) kinase-mediated signal transduction cascade, arrest their cell cycles in G1 phase and grow in a polarized fashion towards the pheromone secreting cells, forming a characteristic cell shape termed a 'shmoo' [7]. Several hundred genes change expression during this process [8]. Shmoos of opposite mating type fuse, producing a diploid organism. The pheromone-response MAP kinase cascade is broadly conserved across eukaryotes, yet characterization of even this canonical signal transduction pathway is incomplete. Here, we describe the development of spotted cell microarrays and their application in defining genes controlling the response of yeast cells to mating pheromone.

**Figure 1:**
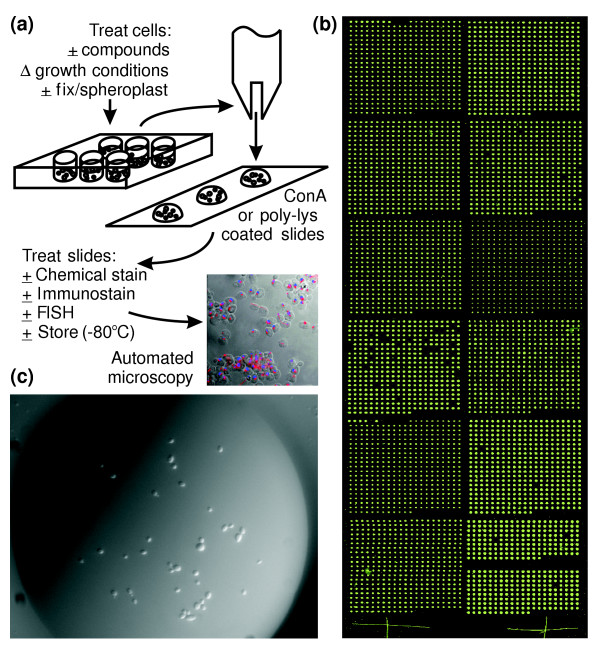
An overview of spotted cell microarrays. **(a) **Cell chips are constructed using slotted steel pins to print cells robotically from 96-well plates onto poly-L-lysine or Con A/Mn^2+^/Ca^2+^-coated glass slides. The sample image shows arrayed yeast cells immunostained for tubulin using fluorescein isothiocyanate (FITC)-conjugated-goat anti-rat IgG/rat anti-α-tubulin (red), overlaid on a bright field image and a DAPI-stained image (blue) of the cells' nuclei. FISH, fluorescence *in situ *hybridization. **(b) **Wide-field light scattering image of a cell microarray (approximately 2 cm × 6 cm) containing around 4,800 viable, haploid yeast deletion strains. The bright dots arise from light scattered when scanning the array with a Genepix DNA microarray scanner. Spots are around 200 μm in diameter, separated by 410 μm. **(c) **Close-up of a typical spot from the microarray showing distinct cells at 40× magnification. This image was taken immediately after printing, so growth medium (YPD, 17% glycerol, 200 mg/l G418) is still visible.

We developed spotted cell microarrays for highly parallel, high-throughput analyses of cell phenotypes, complementing efforts for assessing cell growth and morphology [9-15], protein expression levels [9,16-18], and imaging of tissues [19] and single cells [20,21]. Spotted cell microarrays are distinct from transfected cell microarrays [22-24] or RNA interference (RNAi) microarrays [25], in which mammalian cells are cultured over a microarray spotted with defined DNAs, allowing transfection of the overgrown cells with different clones. Instead, spotted cell microarrays are made by contact deposition of suspensions of cells from an arrayed library onto coated glass slides using a microarray robot (Figure 1). Printing cells directly allows cells of different genetic backgrounds to be arrayed, taking advantage of strain collections such as the set of around 4,800 haploid yeast deletion strains (in which each strain lacks the coding sequence of a single gene) [26,27].

High-density cell microarrays, with each spot containing cells from a distinct deletion strain and all of the strains represented on a single microscope slide, simplify automated image collection and minimize reagent use when probing the cells. With this approach, a single cellular feature can be examined in all approximately 4,800 genetic backgrounds, identifying genes contributing to that feature, associating genes with specific phenotypes, and providing information about spatial structures controlled by the genes. We have successfully created cell chips from 4,848 yeast deletion strains, automated collection of around 20,000 microscope images per cell chip, constructed the initial computational infrastructure to support the microscopy, and used cell chips to screen for genes affecting normal cellular morphology and for genes affecting the response of yeast to mating pheromone.

## Results

### A high-throughput screen of yeast cellular morphology

Cells from each of the 4,848 distinct haploid yeast deletion strains, grown in rich media (YPD), were printed onto glass microscope slides coated with poly-L-lysine or concanavalin A (ConA) using a custom-built high-speed robotic arrayer that is normally used to manufacture DNA microarrays [28]. Figure 1b shows an image of a cell microarray printed using this methodology. Each spot normally contains around 20-40 cells from a single deletion strain, as seen in Figure 1c using a standard microscope. Our preliminary data indicate that arrayed cells remain viable and physiologically normal after printing and washing, although cells are typically fixed for imaging purposes. A cell chip is analyzed using an automated fluorescence microscope to sequentially autofocus and image each spot.

**Figure 2:**
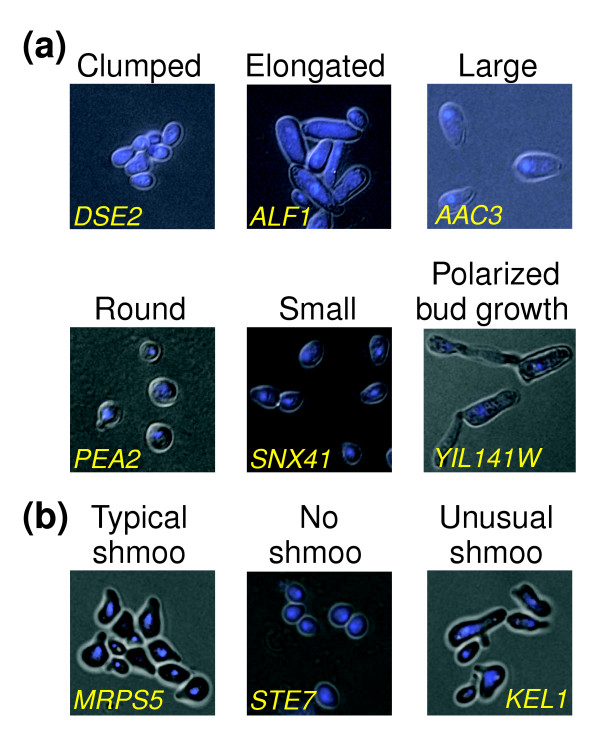
Characteristic yeast cell phenotypes observed on arrays. DIC images from cell chips were collected automatically at 60× magnification with DAPI-stained nuclei superimposed in blue pseudocolor. Each gene name indicates the corresponding deletion strain (for example, *dse2 *Δ*KanMX4 *). **(a) **Six phenotypic classes observed among the haploid yeast deletion strains. *YIL141W *overlaps the *AXL2 *gene, whose disruption in the deletion strain probably provides the observed morphology. **(b) **Changes in cell morphology observed after treating the deletion collection with mating pheromone. Many mutants, such as the *mrps5 *Δ*KanMX4 *deletion strain (left), form 'wild-type'-like mating projections upon adding alpha factor, while cells lacking *STE7 *(middle) fail to form mating projections, and cells lacking *KEL1 *(right) form mating projections of unusual morphology.

As an initial proof-of-concept, we first performed genome-wide differential interference contrast (DIC) imaging to examine the effects of deleting each yeast gene on basic aspects of cellular morphology such as cell shape, size, budding pattern and clumping, from which we expected to find genes controlling fundamental cell growth processes. Systematic analysis of the haploid yeast deletion strain phenotypes on two slides (around 10,000 images) reveals that about 2,000 of the 4,848 strains exhibited atypical morphologies of varying degree. Two independent graders manually assigned numerical scores to phenotypes by severity, penetrance in the population, and type (large, small, elongated, round, and clumped [27], as well as polarized bud growth and pseudohyphal-like morphology). Control experiments were performed by constructing cell chips from known morphology mutants and wild-type strains, and grading these in the same grading scheme. Of these deletion strains, 381 (8%) were considered to have severe morphology defects (Figure 2) to a degree considered significant in the control experiments, with an estimated precision of 82% and recall of 26% (see Additional data file 1).

Genes deleted from strains with an observed morphology defect were often functionally diverse. Nonetheless, certain general functions were enriched, which we evaluated by comparing the sets of strains exhibiting a given phenotype with the sets of strains previously known to exhibit characteristic cell morphologies [27] or with sets of genes associated with distinct Munich Information Center for Protein Sequences (MIPS) functions [29] or Gene Ontology [30] functions. Elongated strains were enriched (*p *< 0.01, as calculated using FunSpec [31]) for genes operating in nucleic acid metabolism, cell-cycle defects, transcription, and meiosis; large strains were enriched for transporter defects; round strains for cell wall, budding, cell polarity, and cell-differentiation genes; small strains for mitochondrial, carbohydrate metabolism, and phosphate-transport genes; and strains with polarized bud growth defects for budding, cell polarity, and filament-formation genes. Large and elongated strains significantly (*p *< 0.01) overlapped strains previously identified with these phenotypes during analysis of the homozygous diploid yeast deletion strains [27]. Additional data files 2 and 3 summarize the morphological defects and functional enrichment, respectively.

### Systematic identification of genes controlling mating-pheromone response

Having established the typical morphology of each haploid deletion strain, we examined the primary morphological differentiation pathway in budding yeast - the response of the cells to the mating pheromone alpha factor during sexual conjugation.

Although this pathway is well studied [7], it has yet to be analyzed to completion. We reasoned that additional genes affecting the pheromone-response pathway, either directly or indirectly, could be identified by examining shmoo phenotypes when the deletion collection was treated with alpha factor. We treated the entire mating type **a **haploid yeast deletion collection with alpha factor, then constructed and imaged spotted cell microarrays from the treated and fixed cells. Two graders manually examined the cell images for the absence of shmoos, grading the images on a numerical scale. Consistency between graders was high, and no systematic grading differences were apparent (see Additional data file 1). Defects in shmooing were found in 142 strains; these either formed no shmoos or formed barely detectable shmoos in the imaged fields of cells (Figure 2b and see Additional data files 2 and 4).

These 142 strains represent a mixture of genes participating in the pathway and false-positive results in the large-scale screen, primarily arising from stochastic sampling of cells from image fields with limited penetrance of shmoos and from ambiguity in identifying cells with mating projections. In practice, we explicitly included ambiguous cases for later retesting, thereby increasing the false-positive rate of the genome-wide screen but decreasing the false-negative rate (see Additional data file 1). We filtered this set for reproducible shmoo defects by manually retesting the 142 strains twice via alpha factor addition (to both mid-log and late-log phase cells) and microscopic imaging; 54 of the 142 strains showed consistent shmoo defects. Of these strains, ten were previously identified as diploid or MATalpha strains in the MAT**a **haploid strain collection (A. Tong and C. Boone, personal communication), which correctly appear insensitive to alpha factor in this screen. Removing these strains (accounting for all diploid and MATalpha contaminants) and six strains whose deletions could not be confirmed by PCR or whose phenotype failed to reproduce in a reconstructed strain (see Additonal data file 1) leaves 38 MAT**a **haploid strains reproducibly defective in shmoo formation. Note that deletion of one of these genes, *GPA1 *, is thought to be lethal except in the presence of additional pheromone pathway mutations [32], implying either strain-specific viability or additional suppressor mutations in the library strain.

### Independent validation of mating-pheromone response genes

**Figure 3:**
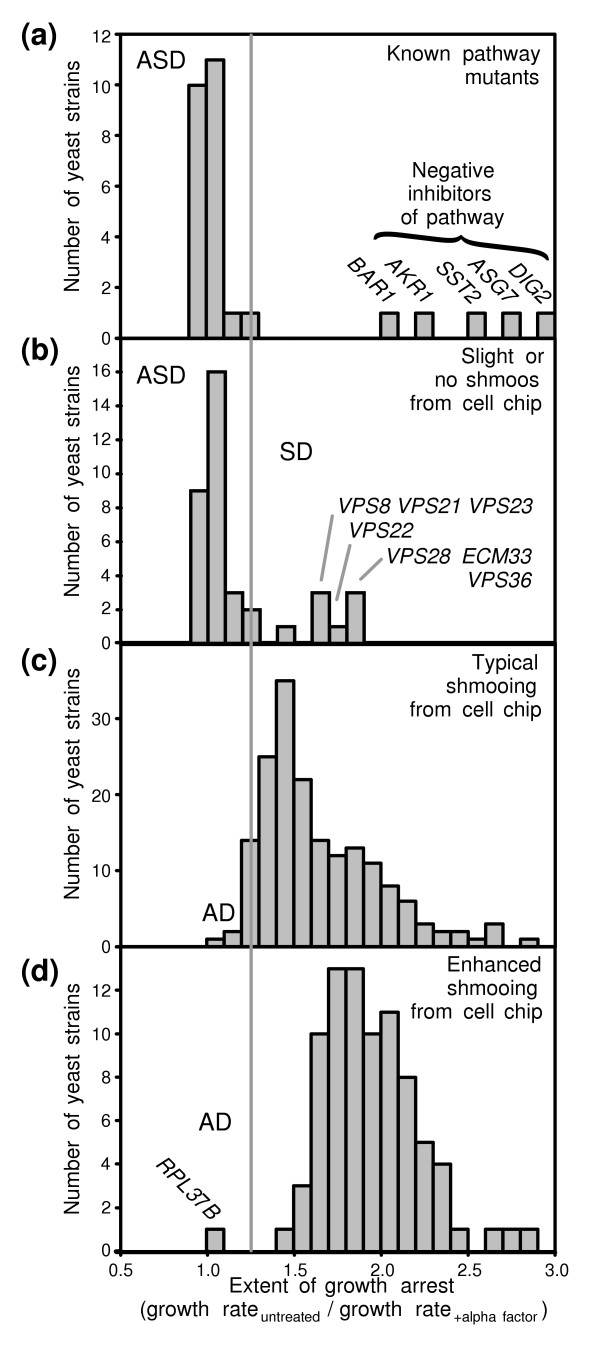
Results of a cell microarray-based genome-wide screen for genes participating in the mating-pheromone response pathway. Strains defective in the pathway fail to arrest growth when treated with alpha factor, unlike wild-type cells. The histograms report the average results of two or three replicate growth assays for **(a) **28 strains containing deletions of genes known to participate in pheromone response, **(b) **38 strains identified from cell microarrays as failing to shmoo properly, **(c) **178 strains forming typical shmoos, and **(d) **91 strains forming shmoos with a notably enhanced frequency in the cell population. The true-positive alpha factor-response pathway mutants (ASD, arrest+shmoo defective) are well separated from non-pathway mutants. Additional mutant categories identified were those defective only in the shmoo pathway (SD, shmoo defective), and those defective only in the growth arrest pathway (AD, arrest defective). Gene names indicate strains deleted for the corresponding genes.

To validate the involvement of these genes in the pheromone-response pathway, we followed up the high-throughput cell-chip screen by conducting growth assays measuring the tendency of the strains to arrest growth upon pheromone exposure. We tested the complete set of 142 deletion strains (that is, the 38 reproducibly defectively shmooing strains and the remaining strains whose defects failed to reproduce) plus 271 additional deletion strains as controls with either normal shmooing (wild-type like, as determined from the cell microarray screen) or enhanced shmooing (marked by increased frequency of shmoos in the cell population), as well as strains deleted for 28 of the 41 genes previously known to be involved in the pheromone-response pathway. The positive controls are clearly differentiated from the normally shmooing strains in this assay (Figure 3), except for those deleted for five inhibitors of the pathway that arrest growth strongly in this assay (that is, they fail to show defective growth-arrest phenotypes). These include strains deleted for *BAR1 *, the protease that degrades mating pheromone [33], and *DIG2 *, which inhibits pheromone-responsive transcription [34].

Figure 3 shows that 30 of the 38 reproducible shmoo-defective strains fail to arrest growth upon exposure to alpha factor to an extent comparable to the positive controls. Lack of growth arrest agreed well with reproducible shmoo defects. These strains were defective in both shmoo formation and growth arrest, implicating the deleted genes in the pathway. An additional four MAT**a **haploid strains first identified as shmoo defective, but not among the 38 reproducibly shmoo-defective strains, also fail to arrest growth upon exposure to alpha factor, implicating the deleted genes in the pathway (see Additional data file 4). Enhanced shmooing strains arrest even more strongly and appear systematically hypersensitive to the pheromone (Figure 3). Thus, the extent of growth arrest in this assay correlates well with the penetrance of shmooing across the populations of cells as measured with the cell-chip assay.

### Comparison with known pathway implicates new genes in pheromone response and shmoo formation

**Figure 4:**
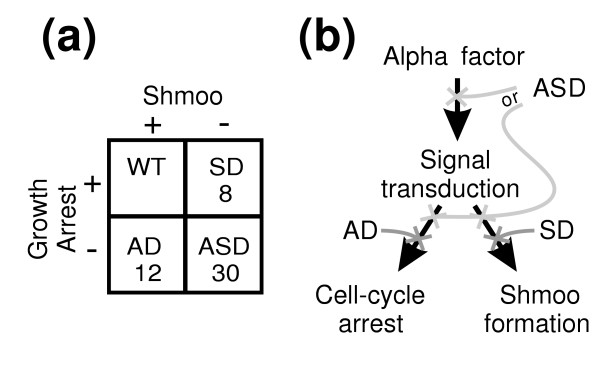
Summary of cell-chip/growth assay results. With two phenotypic screens, we expected three classes of mutants: true-positive alpha factor-response pathway mutants (ASD), those defective only in the shmoo pathway (SD), and those defective only in the growth arrest (AD). **(a) **The number of genes identified in each category; **(b) **their interpretation. Only 413 strains were tested by growth assay, so the number of strains with wild-type phenotypes (WT) is omitted.

As two distinct, although correlated, phenotypes were assayed (growth arrest and shmoo formation), we expected to find genes defective in either or both pathways - a defect in both implicates the gene in the initial alpha-factor response pathway or in both downstream pathways, whereas a defect in only one implicates the gene in the corresponding downstream pathway (Figure 4). We first investigated mutants exhibiting both defects (termed ASD, for arrest and shmoo defective), implicated in pheromone detection and signaling.

**Figure 5:**
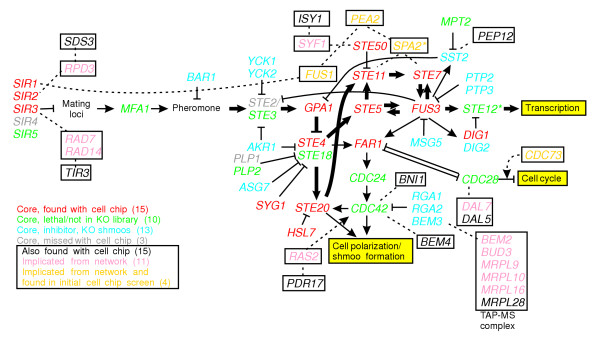
Comparison of results with the known response pathway. This comparison reveals that of the 18 known genes expected to be found in this screen, 15 were recovered (red labels); three core genes were missed (gray labels). Thirteen genes are pathway inhibitors (blue labels) whose corresponding deletion strains shmoo. Ten known pathway genes are absent from the deletion collection (green labels). Of the 15 putative additional genes found, nine (black labels, boxed) could be associated with the core pathway via protein interactions or mRNA coexpression with intermediates (pink labels, boxed). Four network-implicated intermediates (orange labels, boxed) were also found in the initial cell-chip screen, though not reconfirmed. Bold arrows mark the canonical signal transduction cascade leading to transcriptional changes. Thin black arrows indicate activation; barred lines indicate inhibition; dotted lines indicate functional genomics linkages [40]. Genes with asterisks are also implicated in filamentous growth [54].

Comparison with the known pathway (Figure 5, see also [7]) shows that of the 41 genes previously known to be in the pathway, 15 were recovered in the cell microarray experiment. Examination of the remaining genes is revealing: ten genes are not represented in the deletion library (many are essential), 13 genes are inhibitors of the pathway and are thus not expected to be observed in either screen, as the deletion strains still shmoo, and the remaining three genes were missed for technical reasons related to image focus or low cell count. Thus, of the 31 genes expected to be found in this screen, 15 (48%) were correctly identified, including components of the receptor-coupled heterotrimeric G protein (*STE4 *, *GPA1 *), the MAP kinase signal transduction cascade (*STE20 *, *STE11 *, *STE5 *, *STE7 *, *FUS3 *, *FAR1 *, *STE50 *), and silencers of mating loci (*SIR1 *, *SIR2 *, *SIR3 *). Recognizing that negative regulators may not be found in this screen raises the recovery rate to 15/18 genes, or 83%. Interestingly, strains with deletions of certain negative regulators such as *HSL7 *and *DIG1 *are shmoo defective and we correctly identify them in the screen (Figure 5).

Beyond the known signal transduction pathway, 15 genes were found that fail to shmoo and fail to arrest growth upon exposure to alpha factor. Examples include genes with clear functions in polarized growth (*BEM4 *and *BNI1 *), as well as regulatory functions (the histone deacetylase *SDS3 *and the ubiquitin protein ligase *UBR2 *). We separately validated the *BNI1 *and *UBR2 *involvement by reconstructing and retesting the deletion strains. Other strains were PCR-confirmed for the identities of the deleted genes, but not reconstructed (see Additional data file 1), and thus should be validated by strain reconstruction before confirming the definite involvement of these genes in the pheromone-response pathway. There is a general implication of genes affecting membrane properties, including *PDR17 *, which controls phospholipid synthesis/transport [35] and *LAS21 *, which controls glycosylphosphatidylinositol-linked protein transport/remodeling [36]. Several genes encoding plasma-membrane transporters are identified (*QDR2 *and *DAL5 *), as well as a cell-wall biosynthetic enzyme (*YEA4) *and mannoprotein (*TIR3*). Loss of any of these genes disrupts pheromone response, possibly indicating membrane properties feeding back into control of mating response, consistent with the important role of plasma-membrane reorganization in shmooing [37].

Such comparisons with known and literature-associated pathway components, as well as strain reconstructions, allow us to estimate the false-positive rate of this screen. Of the 40 original genes (after removing MATalpha, diploids, and strains not verified by PCR), 15 are known pathway components, three (*BEM4 *, *ISY1 *, *SDS3 *) can be reasonably implicated in polarized growth and pheromone response from literature, two (*BNI1 *, *UBR2 *) were confirmed with reconstructed strains, and two were eliminated as false positives in reconstructed strains. Therefore, 20 of 40 genes were confirmed and two were false positives, placing the false positive rate at 2/22, or 9%. Not considering the three components implicated from the literature raises this to 2/19, or 11%. Nevertheless, as with any genome-wide screen we advise reconstruction of deletion strains before unequivocally concluding that these genes are implicated in the pheromone-response pathway.

Finally, we identified strains defective in only one of the two assayed phenotypes, implicating the genes in downstream pathways. The set of strains that fail to arrest yet shmoo properly (termed AD for arrest defective) was functionally diverse as well as small (in part because only around 8% of the deletion collection was tested for growth arrest - we expect more such mutants given a complete screen for growth arrest). These strains were deleted for *FMP35, RPL37B *, *YHL042W *, *YDR360W *, *YGL214W *, *PUB1 *, *PMT2 *, *TRX2 *, *SFK1 *, *MUP3 *, *SPL2 *, and *STM1*. Conversely, eight genes were identified arresting normally yet failing to shmoo (termed SD for shmoo defective). Interestingly, six of these (*VPS8 *, *VPS21 *, *VPS22 *, *VPS23 *, *VPS28 *, *VPS36 *) are involved in vacuolar protein sorting, with all but *VPS8 *and *VPS21 *specific to class E sorting and resulting in inefficient transport out of the endosome [38], suggesting a critical role of this system in shmoo formation (that is, downstream of pheromone signaling), possibly related to plasma-membrane reorganization [37,39]. The remaining two proteins are involved in polarized growth (*ECM33 *) and transcriptional regulation (the histone acetylase *EAF3 *).

## Discussion

We attempted to connect the 15 putative pheromone-response implicated genes (the ASD set) to the known pathway (the core set) using available functional genomics data by searching for the shortest pathways through protein interaction and mRNA coexpression networks [40] that connected the new genes to the core set. Nine of the new genes could be reasonably connected to the core set by two interactions or fewer (Figure 5), indicating that these genes may have direct, rather than indirect, roles in the pheromone response pathway. As connecting 9 of 15 genes to the core is no more than expected by random trials, these linkages serve only as hypotheses to provide a starting point for experiments validating the associations.

One gene connected in this manner is *SDS3 *, a component of the Rpd3/Sin3 histone deacetylase complex implicated in gene silencing [41], and it is likely that the implication of *SDS3 *in the pheromone response pathway probably stems from the action of this complex on the silencing of mating loci. Likewise, another gene implicated in the screen, the ubiquitin protein ligase *UBR2 *, is an interaction partner of *DOT1 *, a participant in Sir-mediated gene silencing [42], and thus a reasonable inference is that deletion of *UBR2 *may also influence silencing. Another gene from the screen, *ISY1 *, is pleiotropic but connected to control the cell cycle, participating in mRNA splicing and the spindle checkpoint [43]. *ISY1 *exhibits some connection to polarized growth: homozygous diploid deletions of *ISY1 *exhibit abnormal axial budding [44]. Although *MRPL28 *can be connected the core network in this manner, its shmoo defect might also arise by a disruption in the deletion strain of the proper functioning of the adjacent *MFA1 *alpha factor mating-pheromone gene.

Cell morphology phenotypes are rich in information, and although we have focused on strains exhibiting a failure to shmoo, additional strains were identified with morphological defects in the mating projections, such as shown for the *kel1 *Δ*KanMX4 *strain of Figure 2b. We flagged a total of 29 strains producing shmoos of aberrant morphology. These strains, listed in Additional data file 5, are deleted for genes involved in a statistically significant (*p *< 0.01 [31]) fashion in mating, especially for genes of polarized growth (*CDC10 *, *KEL1 *, and *BUD19 *), but also for genes of transcriptional and translational regulation, including components of transcription and chromatin remodeling (*SNF6 *, *SPT3 *, *SPT10 *, *HTL1 *, and *SIN4 *), translational regulation (*CBP6 *, *ASC1 *, and *SRO9 *), and rRNA processing/ribosome biogenesis (*NSR1 *, *RPP1A *, *RPL31A *, *RPS16B *, and *RAI1 *). There is also some interplay between cell morphology and pheromone response phenotypes - for example, the *mrpl28 *Δ*KanMX4 *strain exhibits a large cell phenotype until alpha factor is added, whereupon the cell size defect is corrected, although the cells fail to shmoo (see Additional data file 1).

Interestingly, we also find the extent of alpha factor-induced growth arrest appears largely uncorrelated with the change in expression of the corresponding genes following alpha factor treatment in wild-type cells [8], even for known genes in the core pathway (see Additional data file 1). Instead, the known pathway genes fall into two categories: those whose deletion strains show strong alpha factor-induced growth arrest or those that fail to arrest. The former category is exclusively composed of inhibitors of pheromone-response components. The majority of known pathway genes do not change expression following alpha factor treatment [8], nor do the majority of new genes implicated in the pathway by the combined cell chip/growth inhibition assay. Therefore, the cell chip-based screen complements the information available from DNA microarrays.

## Conclusion

In conclusion, we describe a new genomic-scale technology for microscopy on genetically distinct cells, applied here to measuring the cell morphologies of yeast in the haploid deletion strain collection and to the mapping of genes participating in the response of yeast cells to mating pheromone. Although this paper focuses on cell morphology, cell chips have utility beyond this and can in principle be extended to any organism or cell type for which defined libraries of cells can be arrayed, such as other easily manipulated organisms, banks of bacteria, and deletion libraries for other microorganisms. We expect that diverse collections of strains can be arrayed, such as yeast strains in which proteins are tagged with green fluorescent protein [45]. Just as it proved possible to identify pathways modulated by alpha factor, it should be possible to quickly identify mutants and pathways differentially affected by drugs. A major advantage of the cell chips is the minimal use of expensive reagents on the chips, achieved by limiting the use of antibodies and dyes to single microscope slides, as compared to the approximately 50 96-well plates required to image the complete deletion collection.

The key principle distinguishing cell chips from other approaches (such as immunoassays in 96-well plates) is, however, the separation of cell growth from imaging. Thus, we anticipate the strongest advantage of cell chips will be their use for analyzing the localization of proteins or RNAs by high-throughput *in situ *hybridization and antibody-based immunoassays. Consider the case of printing multiple identical cell chips, but probing each with a different set of dyes or antibodies. Each slide then becomes a unique assay for the dye or antibody target across the set of genetically distinct strains. In this mode, cells from the deletion strain collection are fixed, spheroplasted, and spotted onto microarrays, effectively separating the growth of the cells from the imaging process (a strategy difficult to achieve with plate assays). Around 200 cell chips can be made in a single printing session; each serves as a separate imaging assay when probed with an antibody to a distinct target, revealing the change in localization and expression of that target across the approximately 4,800 genetic backgrounds. The resulting images would indicate synthetic genetic interactions between the probe targets and the deleted genes, and the act of imaging becomes a scaleable, easily replicated assay on standardized cell chips for the high-throughput generation of synthetic interactions. Combining cell-chip throughput with automated image processing [12,46,47] should provide quantitative strain- and gene-specific data. Data from such experiments will generate functional and statistical connectivities between genes [48], ultimately leading to comprehensive network analyses of genes [49].

## Materials and methods

### Cell microarray construction and imaging

All methods are described in full in Additional data file 1. In brief, cell microarrays were constructed by contact deposition of suspensions of yeast cells from the arrayed collection of *S. cerevisiae *haploid deletion strains (BY4741 genetic background; *MAT ****a ****his3 *Δ*leu2 *Δ*met15 *Δ*ura3 *Δ) onto Con A [50] or poly-L-lysine-coated glass slides using a custom-built DNA microarray printing robot [28]. In about 12 h, more than 100 slides can be printed, each containing the entire deletion collection as well as the isogenic wild-type parent strain as a control. Cell arrays may be used for imaging immediately after printing or stored at 4°C or -80°C, provided that the cells are printed with glycerol. Centrifugation enhances adherence of cells to the slide, permitting washing before staining and imaging. Cell images were collected via automated microscopy, using a Nikon E800 microscope with computer-controlled *X-Y *stage and piezoelectric-positioned objective, by scanning to the position of each spot, autofocusing, and capturing the image with a Coolsnap CCD camera (Photometrics, Tucson, USA). Images were stored in a custom cell microarray image database (Cellma) [51] for manual examination or automated image analysis. Using Perl scripts and custom MetaMorph (Universal Imaging Corporation, Sunnyvale, USA) journals, a full set of approximately 5,000 images can be collected from a slide in bright-field mode in less than 4 hours or for fluorescent images in around 10 h. Control cell chips, grading schemes, and morphology analysis details are described in the Additional data file 1.

### High-throughput screen for strains unresponsive to alpha factor

To examine cell morphology after stimulation with alpha factor, each yeast deletion strain was subcultured into fresh YPD medium in 96-well Costar tissue culture plates (Corning, Corning, USA), grown for 36 hours at 30°C, centrifuged, and washed with YPD, pH 3.5, to inactivate the Bar1p protease [52]. Alpha factor (350 μg/ml) was added to each sample well, a concentration measured by titration (as in [53]) to induce shmoo formation in around one half of the cells in the majority of deletion strains (see Additional data file 1). Cells were incubated for 4 hours at 30°C, fixed with 3.7% formaldehyde for 1 hour at room temperature, washed with YPD containing 17% (w/v) glycerol, supplemented with 20 mM CaCl_2_, 20 mM MnSO_4_, then spotted onto Con A-coated glass slides. Slides were stained with DAPI, imaged by automated microscopy, and manually scored by two independent graders for extent of shmooing.

### Assay of alpha-factor-induced growth arrest

Four hundred and twenty-six selected deletion strains were grown overnight in YPD, centrifuged, and washed with YPD, pH 3.5 [52]. The cultures were split into replicate 96-well plates of YPD, with and without alpha factor at a final concentration of 25 μg/ml, maintaining cells at an optical density at 600 nm (OD_600_) of around 0.2-0.5 [52]. Plates were incubated at 30°C for 10 h, recording OD_600 _hourly from each strain. The slope of each growth curve was calculated from a plot of log(OD_600_) versus time. The effect of alpha factor on the strains was obtained as the ratio of the slope from the untreated sample to that of the alpha-factor-treated sample. Average ratios were calculated from two or three independent assays.

## Additional data files

The following additional data are available online with this paper. A detailed description of methods for constructing, imaging, and evaluating spotted cell microarrays is included as Additional data file 1. Data relevant to the measured cell morphologies (Additional data file 2), computational analysis of enriched functions (Additional data file 3), pheromone growth-arrest phenotypes (Additional data file 4), and lists of implicated genes (Additional data file 5) are available. All spotted cell microarray image data and experimental protocols are available from the Cellma cell microarray database [51].

## Supplementary Material

Additional data file 1 A detailed description of methods for constructing, imaging, and evaluating spotted cell microarrays. Click here for file

Additional data file 2 Measured cell morphologies. Click here for file

Additional data file 3 Computational analysis of enriched functions. Click here for file

Additional data file 4 Pheromone growth-arrest phenotypes. Click here for file

Additional data file 5 Lists of genes implicated in the mating-pheromone response pathway. Click here for file
