# Supplementary material for: Systematic profiling of cellular phenotypes with spotted cell microarrays reveals mating-pheromone response genes
Source: Genome Biol. 2006 Jan 31;7(1):R6. doi: 10.1186/gb-2006-7-1-r6 (PMC1431703; doi:10.1186/gb-2006-7-1-r6)
Supplement: Additional data file 1 — A detailed description of methods for constructing, imaging, and evaluating spotted cell microarrays. [file gb-2006-7-1-r6-S1.pdf]

Supplement, **Systematic profiling of cellular phenotypes with spotted cell microarrays reveals new mating pheromone response genes**

METHODS

Yeast deletion strains and growth conditions Cell microarrays (cell chips) were manufactured containing all the strains from the *S. cerevisiae* haploid deletion collection. This is a set of strains in the BY4741 genetic background (MATa *his3Δ leu2Δ met15Δ ura3Δ*) generated by the international yeast deletion consortium, in which each strain contains a chromosomal gene replacement of a non-essential gene with a selectable KanMX4 marker that confers resistance to the antibiotic G418[26, 27]. We obtained the arrayed library of 4848 such haploid deletion strains from Invitrogen. Frozen cell cultures in 96-well plates were thawed and used to inoculate 96-well Costar tissue culture plates with 200 µl YPD medium containing the antibiotic G418 (200 mg/L) and 17% glycerol, using a Beckman Biomek FX 96-well pipetting robot to perform all pipetting operations. Copies of the strain collection were incubated for growth at 30 C, monitoring cell growth by optical density measurement at 600 nm using a 96-well plate reader. Quality control for sterility and cross-contamination was performed by monitoring control wells empty of cells in the master plates. After growth at 30 C for 2 days, copy plates were agitated with a plate shaker, sealed and frozen at -80 C, with each copy thawed prior to use for printing microarrays of cells.

Slide preparation Poly-L lysine or concanavalin-A coated glass Golden seal microscope slides were used for all cell arrays. Poly-L-lysine promotes adherence of yeast cells by electrostatic interactions, while the lectin ConA binds to mannose residues in the yeast cell wall. Poly-L lysine coated slides were prepared with an identical protocol to that standardly used for DNA microarrays, essentially coating with a 1 mg/ml solution of poly-L-lysine in phosphate buffered saline (PBS), then rinsing in deionized water. Concanavalin-A-coated slides were prepared by incubating slides 15 minutes in a 0.1 mg/ml solution of ConA (Sigma) in PBS[50], then washing briefly with 95% ethanol. Only freshly prepared slides were used for each print. Use of ConA coated slides also required addition of 20mM $CaCl_2$ and 20mM $MnSO_4$ to each well of cells (typically, added to fixed cells) in order to activate the ConA for binding (Figure S1).

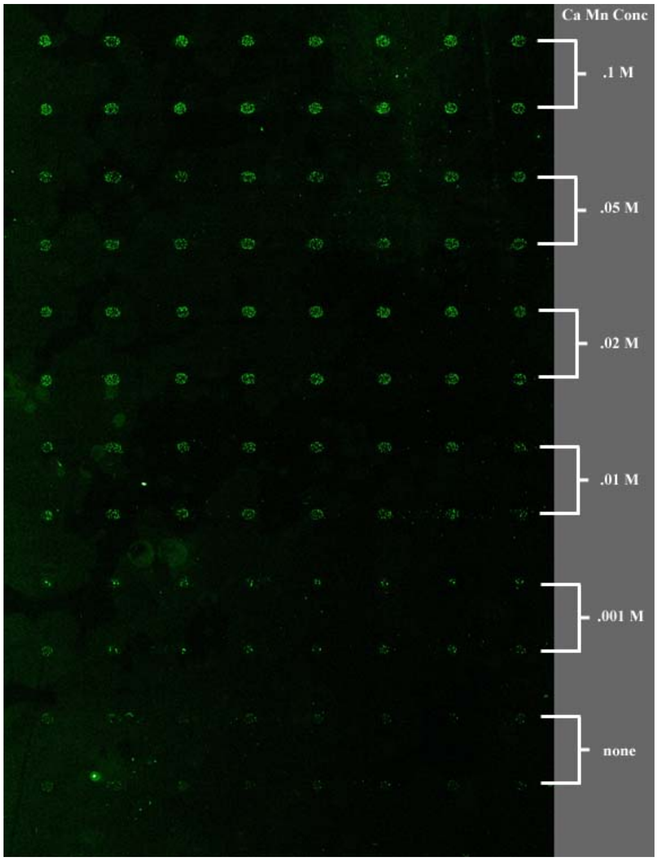

Figure S1: Yeast cell adherence to a ConA-coated cell microarray slide surface as a function of Ca/Mn concentration. Cells were spotted, then washed, to test adherence.

Printing of cell microarrays Cell microarrays were printed by contact deposition of suspensions of yeast cells from the thawed arrayed strain collection onto coated glass slides using a custom-built DNA microarray printing robot (Figure S2). Printing was carried out using conically tapered 1/16 inch diameter stainless steel printing tips with 0.0015 inch slots (Majer Precision Engineering; MicroQuill 2000) that are sterilized between print runs. The resulting spots are ~200 µm in diameter, spaced 410 µm apart; spots were printed in 12 blocks, each 21 spots in width. In a standard print run, the tips are rinsed and vacuum dried 3 times after each loading and printing step, sufficient to prevent carryover of cells from one well to the next, as judged by microscopic inspection of putatively empty spots. Thawed 96-well plates with cell suspensions are agitated gently just prior to placement on the printing robot to ensure a mixed cell suspension. Plates are kept covered at all times before and after printing. During printing, the microtitre plates are kept under a clean acrylic cover at all times except during pick up of the cells. All surfaces, including the vacuum slide platter and the underside of the acrylic dust cover are sterilized by wiping with 70% ethanol. In our experience, these procedures ensure that there is no detectable

contamination of wells in the plates or of spots on the microarray. After printing, the slides are centrifuged flat at 1500 x g for 5 minutes in a swinging bucket centrifuge adaptor to promote the adherence of the cells to the slide surface. Cell microarrays can be imaged immediately at this point or stored at 4 C or -80 C for extended periods of time. To prevent condensation when thawing slides stored at -80 C, frozen slides were rapidly thawed by dipping briefly in room temperature 95% ethanol, then centrifuged dry in an empty 50 ml conical tube at 600 rpm for 5 minutes.

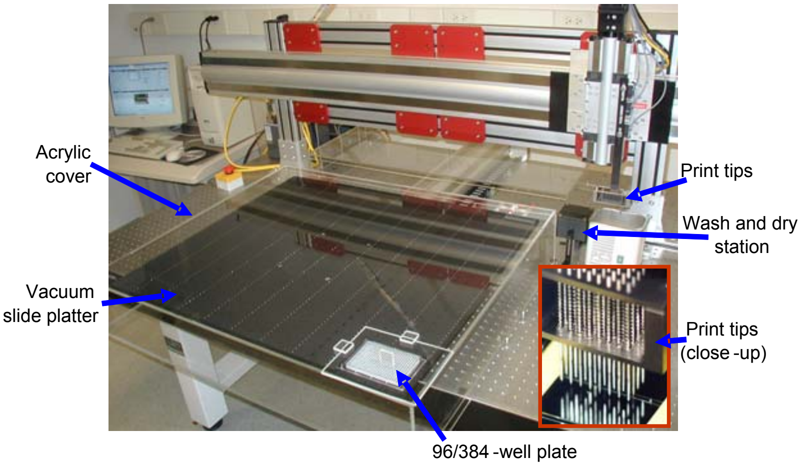

Figure S2: Custom-built microarray printing robot

Scanning of cell microarrays Cell microarrays were imaged in two steps: first, the lattice of cell spots was determined using a standard DNA microarray scanner, then each spot was imaged using an automated microscope. Prior to staining and imaging, each slide is marked with four reference marks using a diamond scribe (two of these marks are visible at the bottom of the image in Figure 1), then the slides are scanned using an Axon GenePix 4000A/B microarray scanner. The spots of cells are detected as bright spots in the 532 nm detector channel because of light scattering by the cells and by the droplet of media or dried liquid at each spot (see Figure 1). Glycerol present in the medium that cells are suspended in during printing inhibits evaporation and enhances the brightness of each spot. GenePix scanner software is then used to fit a two-dimensional grid over the spots to define the block, row, and column location of each spot, thus providing an *x, y* coordinate with each spot in the scanner's system of coordinates. These *x, y* coordinates are written out to a GenePix GPR-format file, as well as the associated strain identities (stored as a GenePix Gene Array List (GAL) file). At this point, each slide has an associated set of coordinates describing the relative locations of each cell spot, their identities, and the locations of the reference marks. Spot coordinates can be converted from the GenePix coordinate system to the optical microscope coordinate system through the use of the four reference points and an affine transformation. Slides are then stained or otherwise manipulated prior to microscopy. For typical brightfield or DIC microscopy, slides are washed with water after scanning in order to remove glycerol, dried *via* 5 minutes of centrifugation, and a few drops of mounting media are applied containing 100ng/ml DAPI nuclear stain. Slides are then covered with 24x60mm cover slips and sealed with nail polish.

Automated microscopy was carried out using a Nikon E800 with the CF160 optical system, and outfitted with a motorized X-Y stage with 0.1 micron resolution, a piezoelectric auto-focus device for 9.7 nm focusing resolution, a Photometrix Coolsnap camera with 1392x1040x12 bit pixel resolution, filters for Differential Interference Contrast (DIC), fluorescence, and visible wavelengths, and MetaMorph software. First, the reference marks on the slide are found and their positions recorded using the microscope's coordinate system. An affine transformation matrix is derived that converts coordinates in the GenePix coordinate system to that in the microscope coordinate system, then applied to all points in the GPR file output from the GenePix scanner, creating a MetaMorph format STG file containing the coordinates of all spots converted into the microscope's coordinate system. Images were collected at each spot by executing a MetaMorph 'journal' macro at each spot listed in the STG file that auto-focused and captured brightfield and fluorescent images, saving each image in TIFF and JPEG format. An entire slide with ~5000 spots can be imaged in ~10 hours, capturing both fluorescent and DIC/brightfield images.

Image annotation database We developed an online relational database for warehousing and annotation of cell microarray images. This database, called Cellma (for Cell MicroArrays), centers around a suite of web pages driven by a MySQL relational database. The images are stored on a central server. The database administrator creates user accounts, enters information about the organisms, strains, and genes studied using the web interface. For data submission, experimenters first copy images directly to the appropriate location in the directory hierarchy, then create an entry for the slide including name, description, date, preparation,

experiments, treatments, and other information. Cellma currently supports online manual annotation of high throughput microscopy images. Although tedious, phenotypes can be reliably scored by visual inspection of images within reasonable timeframes. One experiment manually graded this way was composed of 5,292 images and took 20 hours to complete. The images were scored for intensity and penetrance of 10 phenotypes and for cell count. Two graders independently scored the images to ensure consistency.

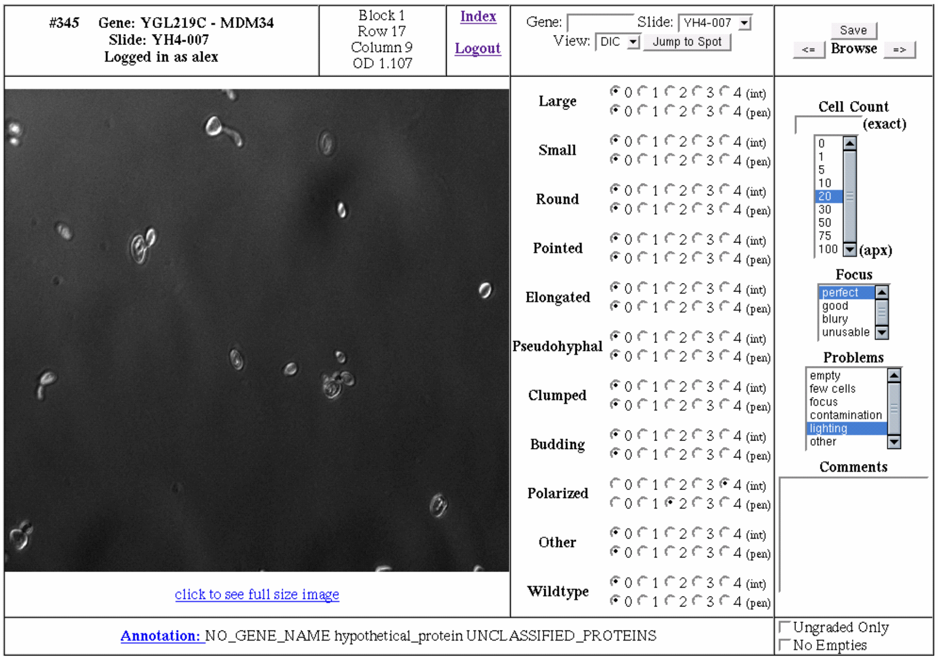

Figure S3: Screenshot of a Cellma annotation database page. Morphology phenotypes for each deletion are assigned using a structured annotation scheme. Phenotypes are classified according to their severity and penetrance on a numerical scale, and are specific for a given spot of mutant cells and a given microarray.

Primary data are stored in the form of image files (one file per spot image) with standardized file names that include the slide name, gene name, cell chip coordinates, and wavelength captured. Files are saved in both TIFF format, for computational analysis, and in JPEG format, for visual inspection. All other data – user accounts, print and slide descriptions, grades – are stored in a relational database using the MySQL relational database management system (RDBMS) running under Linux. The database has been designed for flexibility in anticipation of other organisms, experiments, and analyses. There are currently five publicly available pages for examining images, viewing results of grading, and referencing slide information, as well as private tools for experimenters and administrators, such as a manual phenotype scoring page, a page to set up and execute automated analyses, and interfaces to manage users, prints, and slides.

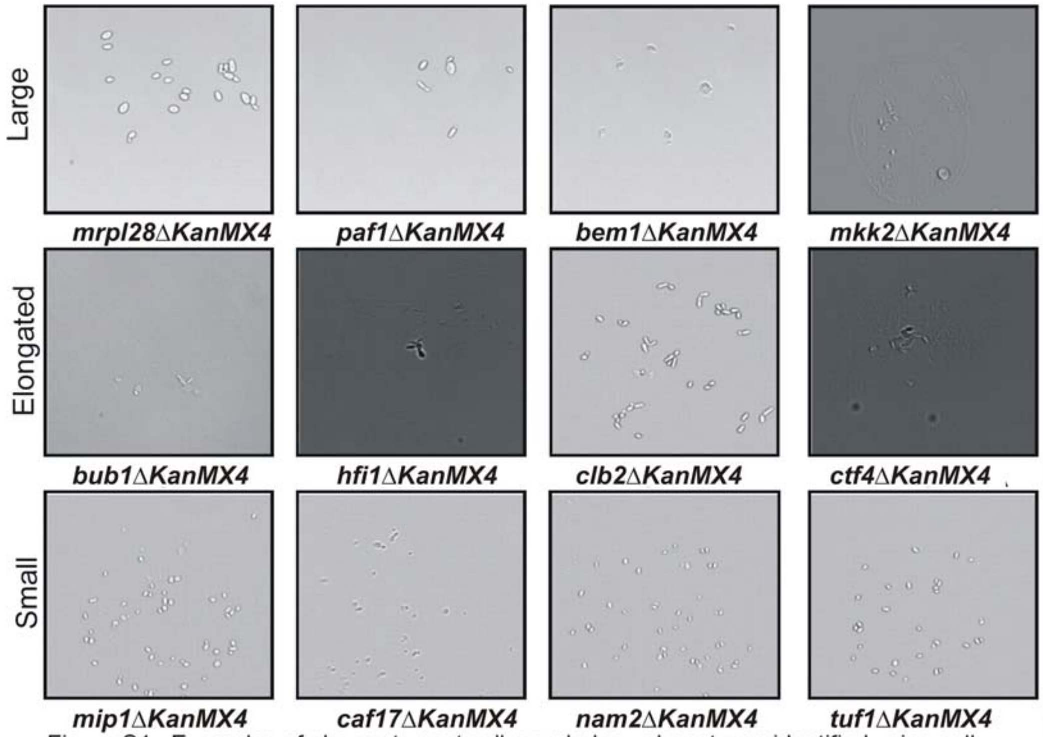

Figure S4: Examples of aberrant yeast cell morphology phenotypes identified using cell microarrays. Each image shows cells deleted for the indicated gene.

For scoring phenotypes, there is a dynamically generated scoring page. Based on experiments applied to a slide and the collected fluorescent wavelengths, the page prompts the grader for intensity and penetrance of each appropriate phenotype (Figure S3). It also prompts for cell count, focus quality, and problems, and allows the grader to enter comments. The database supports easy navigation between genes, image types, and slides. Graders can ignore

known empty spots and can skip spots and return later. Results of scoring phenotypes can be queried by a combination of gene name, experimental procedure, or mutant phenotype using the web interface.

<u>Scoring cellular morphology phenotypes</u> Two graders independently evaluated the set of images from a yeast cell microarray for strains with atypical morphologies. Phenotypes were scored related to *cell size* (mutant phenotypes being *large* or *small* with respect to the wild-type control), *cell shape* (mutant phenotypes being *round*, *elongated*, *pointed* with respect to the wild-type ovoid shape) or either a *pseudohyphal*, *clumped* or *polarized bud growth* or other *budding* defects. In each case, the intensity of a phenotype is assigned a score ranging from 0 to 4, increasing with the severity of the phenotype. The penetrance of the phenotype across the population is scored, ranging from 0 to 4, where 0 indicates absence of the phenotype, and 4 indicates 100% of the cells exhibit the phenotype. Figure S4 illustrates examples of cell morphology phenotypes. Table S1 summarizes phenotypes observed for the mutant strains.

In order to estimate the precision and recall achieved by this manual scoring, a control cell chip of 960 spots was constructed by randomly distributing spots of wild-type cells and known morphology mutants. This slide was scored in a fashion identical to that described above, and the accuracy of graded morphologies was calculated as a function of the sum of the grader-assigned intensities+penetrances (i.e., collapsing grades from the two graders to a single score ranging from 0 to 16). Figure S5 plots the recall and precision calculated from this control experiment as a function of the grader-assigned scores. Given TP = # of true positive grades, FP = # of false positive grades, and FN = # of false negative grades, recall is defined as TP / (TP + FN), and precision is defined as TP / (TP + FP). At a minimum threshold score of 8 (typically requiring agreement by both graders as to the presence of the phenotype to an intermediate degree), the overall set of control mutant phenotypes were identified at 82% precision, 26% recall. Wild-type cells (flagged by the graders as the absence of mutant phenotype) were identified at this threshold at 66% precision, 100% recall. Thus, graders were conservative at this threshold in calling mutant phenotypes, resulting in a relatively high confidence in the mutants identified at this threshold. This threshold was used for the identification of morphology mutants in the full cell chip screen.

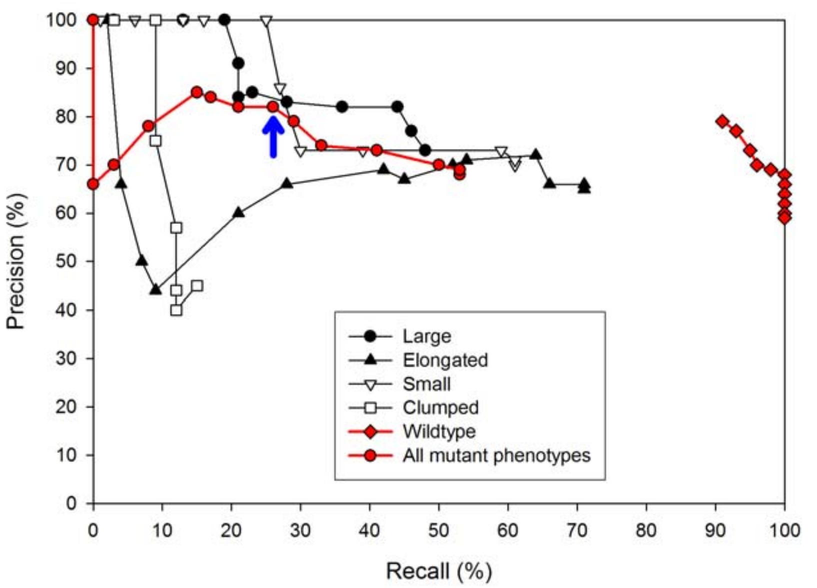

Figure S5: Recall/precision of manual grading of morphology mutant/wild-type control cell chip. The blue arrow indicates the threshold chosen for selection of morphology mutants in the full cell chip screen.

<u>Analysis of cell morphology mutants</u> Strains exhibiting significant morphology defects as defined above were analyzed for functional trends among the deleted genes by searching for statistically significant intersections between the sets of deleted genes giving rise to a particular phenotype with the sets of genes associated with particular Gene Ontology cellular components, molecular functions, and biological processes; MIPS phenotypes, protein complexes, and functional classifications; and cellular morphologies and yeast fitness data previously identified by the yeast deletion consortium[27]. Using the program FunSpec[31], the probability of each intersecting set occurring at random was calculated under the hypergeometric distribution. Significant relationships are reported in Supplemental Table 2 for each set of morphology mutants. Morphology mutants are listed by type in Supplemental Table 4.

Screening for genes affecting the mating pheromone response pathway Using cell microarrays, we identified genes affecting the intracellular signaling pathway used to respond to the yeast mating pheromone. The pheromone response pathway is a signaling cascade that is activated when yeast cells of opposite mating type are in proximity and secrete mating pheromone. When MATa type yeast cells are treated with the pheromone alpha factor, the activation of this pathway causes G1 arrest and a characteristic "shmoo" morphology[6,5]. Defects in this signaling pathway prevent shmooing after treatment with alpha factor. We reasoned that additional protein functions that affect the pheromone response pathway, either directly or indirectly, could be identified by examining cell morphology phenotypes and shmoo phenotypes when the deletion collection was treated with alpha factor. To do this, the yeast deletion collection was grown to saturation in 96 well plates. Each plate was sub-cultured into fresh YPD in 96 well Costar tissue culture plates and allowed to grow for 36 hours at 30 C without shaking. The plates were spun and washed multiple times in YPD, pH 3.5 to inactivate the secreted extra-cellular Bar1p protease[52], which rapidly and efficiently degrades alpha factor. Alpha factor was then added to each sample well. As higher cell densities and different wash conditions require very different alpha factor concentrations[53] (more Bar1p protease may be present, resulting in more degradation of alpha factor and lower effective pheromone concentrations), and because the 96-well plate scale-up of the pheromone response assay placed certain constraints upon the experimental conditions, including higher cell densities, less efficient washing, and certain time constraints (such as a 4 hour incubation time), it was therefore necessary to optimize pheromone concentrations under the precise conditions used in the imaging assay.

We performed titrations of alpha factor under these conditions, measuring the observed percentage of cells shmooing as well as the cell viability (*via* use of the live cell-specific fluorescent stain FUN 1 from Invitrogen/Molecular Probes). Under these

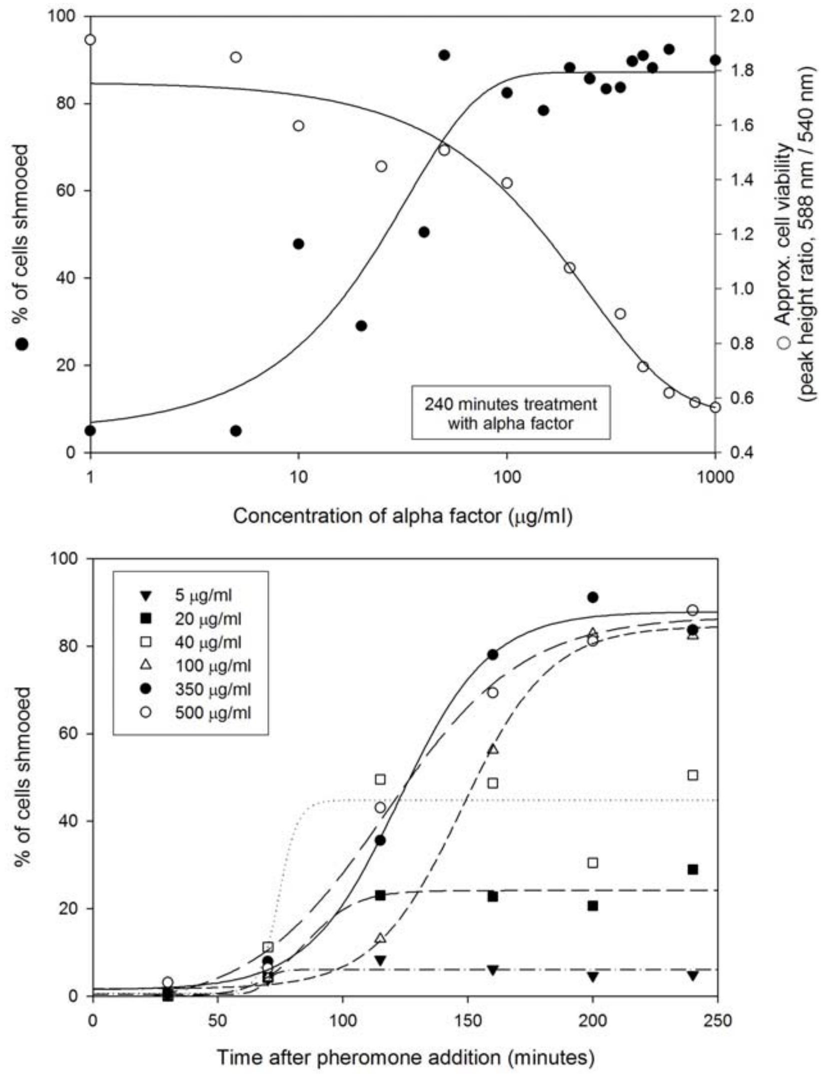

Figure S6: Pheromone concentration (top) and time (bottom) dependence on shmooing

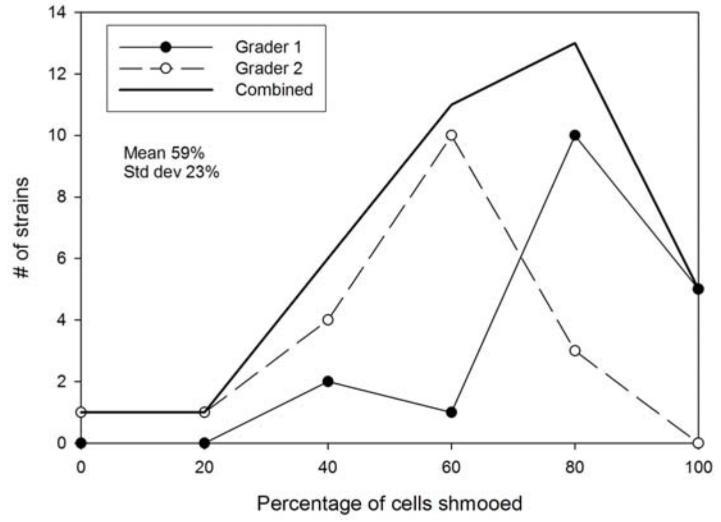

Figure S7: Histogram of percent shmooing of a number of different deletion strains measured on cell-chips

conditions, we find that alpha factor concentrations above 100 μg/ml are required to induce efficient shmoo formation in the wild-type parent strain (Figure S6 top), with some minor variation in the concentration required across a sampling of deletion mutants. Importantly, the fraction of cells shmooing did not decrease as alpha factor concentration increased. In the titration assay, we find evidence for alpha factor-induced cell toxicity at levels >100 μg/ml, although a significant fraction of the cells are still alive at levels up to 300-400 μg/ml. In testing the time dependence of the large-scale assay, we see the percentage of shmoos rise with increasing time, reaching full shmooing only after 200 minutes (Figure S6 bottom). For the full-scale assay, we empirically found that alpha factor concentrations slightly higher than those suggested by the titrations were required to maximize the percentage of detected shmoos; we used 350 μg/ml alpha factor, a concentration sufficiently high to induce shmoo formation in approx. 1/2 of the cells in the majority of the deletion strains (and the wild-type control samples) under these conditions. Because the percentage shmooing on the arrays was lower than expected from the titrations (~59%, as measured across 40 images & shown in Figure S7), it appears that the cells experienced a lower effective concentration of alpha factor than in the titration assays, probably due to less efficient washing on the full large-scale assay, resulting in less Bar1p protease inactivation and therefore more degradation of the alpha factor.

After 4 hours of treatment at 30 C, the cells were fixed in 3.7% formaldehyde for 1 hour at room temperature and washed with YPD containing 17% (w/v) glycerol. At this stage, 20 mM $CaCl_2$ and 20 mM $MnSO_4$ were added to each well. The cells were now spotted onto pre-cleaned glass slides coated with ConA. While the scoring of phenotypes on these alpha-factor treated cell chips was in progress, we also examined shmoo phenotypes of several hand-picked deletion mutants that had previously been identified as cell morphology mutants in our earlier cell microarray analyses. The shmoo phenotype of these mutants were compared to that of wild-type cells as well as cells defective for genes known to have a role in the pheromone response signaling pathway.

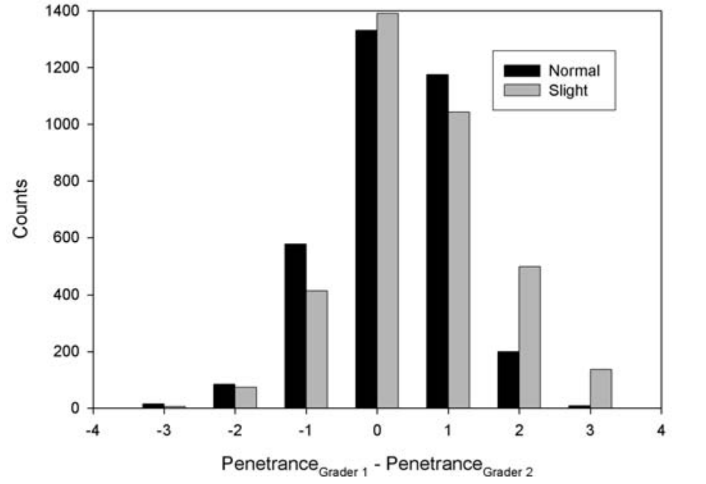

Figure S8: Grader agreement on shmoo phenotypes.

Manual scoring of shmoo defects After imaging the alpha factor-treated yeast cells on the cell chip, two independent graders visually scanned the set of ~5000 images on the following grading system: The intensity of shmoo phenotypes (e.g., the morphology of the cells) was graded by scoring shmoos into 3 categories: Slight shmoo, Normal Shmoo, or Others, referring to the shapes (degree of shmooing) of the alpha factor treated cells, accompanied by a measure of the abundance of that phenotype across the population of cells imaged, ranging from 1-4 (0-100%). For example, for the concentrations of alpha factor chosen, a normal 'shmoo' phenotype (wild type background) had a Normal (2) and Slight (2) indicating that 50% of the cells in the spot had a typical shmoo phenotype, and 50% failed to shmoo. By contrast, a shmoo defective strain would lack any "Normal" or "Other" shmoos, and would be composed of only Slight shmoos; a Normal (4) would indicate an enhanced fraction of normal-looking shmoos in the population, suggesting a hypersensitive response to alpha factor but no change in shmoo morphology. The 'Other' class of shmoos indicated unusual shmoo phenotypes (e.g., see *kel1ΔKanMX4*, Figure 2B), such as from bud neck defects.

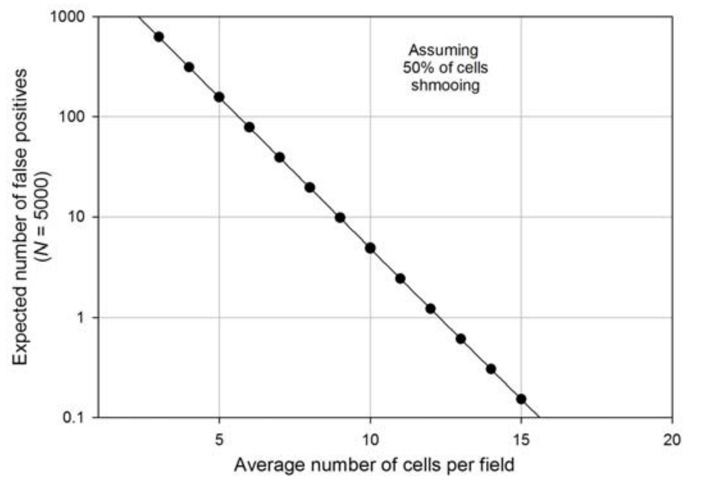

Figure S9: Expected shmoo-less false positive rate.

Figure S8 shows a histogram of the agreement of grades from the two graders—the Gaussian grade distributions indicate that the graders were largely consistent and varied from each other in a stochastic fashion, with no systematic grading bias exhibited, except for an approx. 1/2 unit higher penetrance on average for grader 1 relative to grader 2. Note that for shmoo defective strains, we could estimate from the binomial distribution that we should anticipate a low false positive identification rate (<10 strains) provided that the number of cells per image field was uniformly high (>10 cells/image), as plotted in Figure S9. We expected higher false positive rates from images with low cell counts and from ambiguity in visually determining shmoo phenotypes. In practice, we tended to include such ambiguous cases for manual follow-up, increasing the false positive identification rate of the large-scale screen while lowering the false negative identification rate.

Yeast growth curves +/- alpha factor To test if yeast strains arrested growth in the presence of alpha factor, selected strains were picked from the yeast deletion library and grown in YPD overnight until they attained log phase growth. The cultures were spun and washed with YPD pH3.5 to inactivate Bar1p protease[52]. The cultures were subsequently split into replicate 96 well plates, with and without alpha factor at a final concentration of 25 ug/ml, while keeping cells to an OD600 of ~0.2-0.5, as recommended[52]. The plates were incubated at 30 C for 10 hours without shaking and their absorbance was recorded at 600 nm each hour. The slope of each growth curve was calculated from a plot of log OD600 vs. time. The effect of alpha factor on the strains was obtained as the ratio of the slope from the untreated sample to that of the alpha factor treated sample. Average slope ratios were calculated from 2-3 independent assays. This analysis, in combination with the cell microarray alpha factor treatment analysis, enabled the identification a number of genes whose deletion affected the ability to form shmoos after alpha-factor treatment (Figure S10; Table S3). The growth curves and correct recovery of the known pheromone response pathway genes by the cell chip assay therefore provide two independent validations that the identified genes are relevant to the pheromone response pathway.

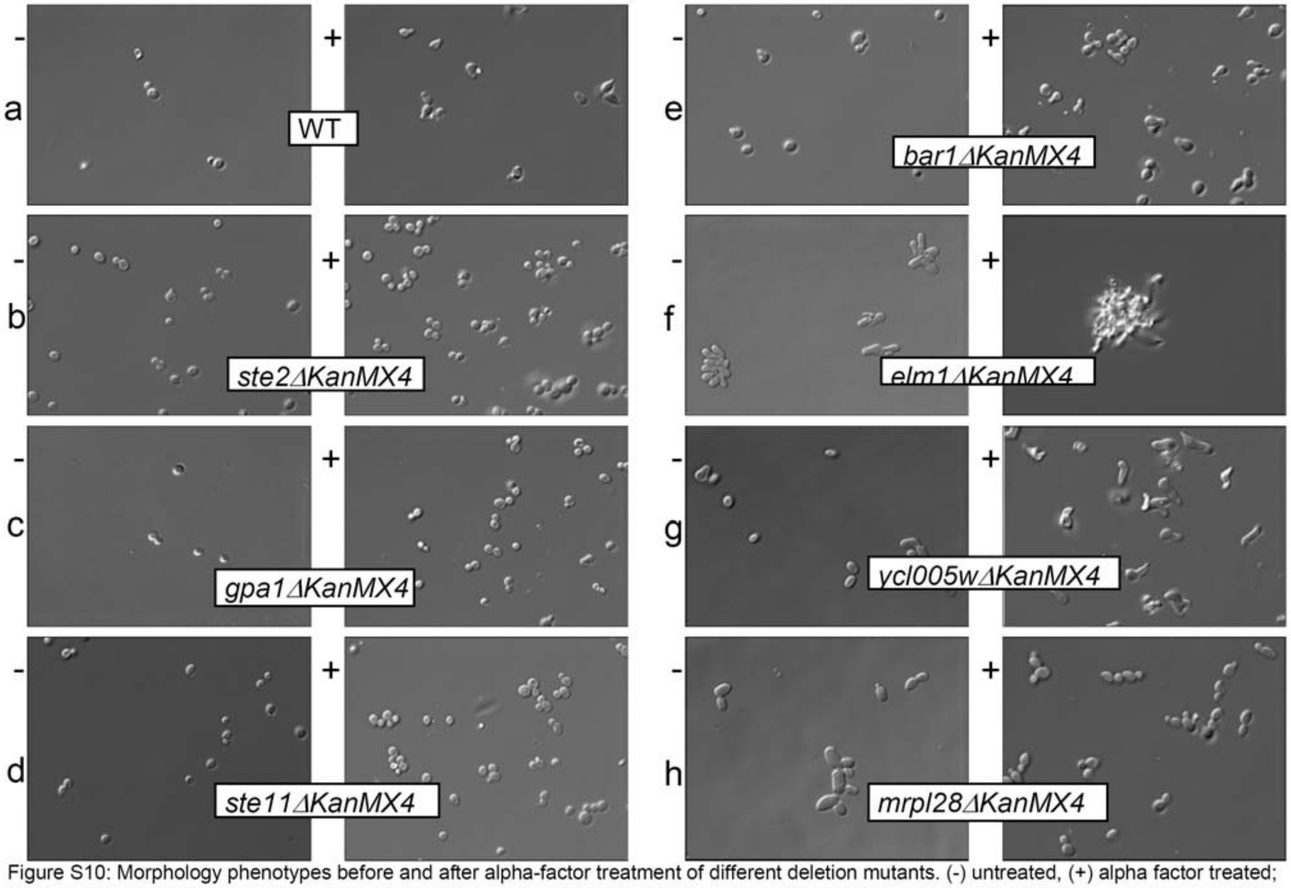

Figure S10: Morphology phenotypes before and after alpha-factor treatment of different deletion mutants. (-) untreated, (+) alpha factor treated; (a) BY4741 parent strain showing ~90% typical shmoos; (b) *ste2ΔKanMX4*, (c) *gpa1ΔKanMX4*, (d) *ste11ΔKanMX4* strains – known pheromone response pathway mutants showing defective shmooing; (e) *bar1ΔKanMX4* strain showing 'wild type' like shmoos used as positive control; (f) *elm1ΔKanMX4* strain, an elongated clumpy mutant showing unusual pheromone response; (g) *ycl005wΔKanMX4* strain (unannotated), a moderately elongated mutant, showing unusual pheromone response; (h) *mrpl28ΔKanMX4* strain, a moderately large mutant and a previously unrelated pheromone response mutant showing defective shmoos.

False positives due to the presence of diploids and MAT alpha strains The systematic deletion strain collection used in our large scale screen was MATa and haploid. This collection is now known to contain a few strains that are actually either diploid or MAT alpha (C. Boone and A. Tong, personal communication). Such contaminating strains would be expected to show up as false positives in our screen because diploid or MAT alpha strains are not sensitive to alpha factor. We removed these known diploid or MAT alpha strains from our list of genes whose deletion affects the response to the mating pheromone alpha factor. We also examined the list of genes whose deletion is known to render MATa yeast cells sterile, based on observations made during large-scale synthetic lethal analysis (C. Boone and A. Tong, personal communication). These sterile strains are incapable of mating, likely because of a defect in the mating pheromone response. Interestingly, 11 out of 15 strains known to be MATa sterile were identified in our cell-chip screen, illustrating the power of this approach to recapitulate known phenotypes.

Polymerase Chain Reaction (PCR) validation of deleted genes The set of 42 deletion strains identified as ASD, SD, or AD, but not known to be in the core mating response pathway, were validated for the identities of their deleted genes by PCR. We performed PCR validation for each of the 42 strains using a PCR primer specific for the kanamycin cassette with a primer specific for the region upstream of the deleted gene (using the ‘KanB’ primer and the 42 gene-specific ‘A’ primers designed by the Yeast Gene Deletion Consortium[27] and using the same protocol as in ref. 52), from which we expected PCR products of approx. 400-700 bp in size if the strain correctly contained the kanamycin insertion cassette and was deleted for the appropriate gene. 38 of the 42 strains (90 %) were confirmed by this approach as harboring the correct deletion. 3 of the strains were not confirmed by either the ‘A’ specific primer/’KanB’ primer pair or by PCR with the corresponding downstream (‘D’) primer and ‘KanC’ primer pair, and thus cannot be confirmed as containing the expected deletion (Figure S11), while 1 strain (*mdm32ΔKanMX44*) gave a larger-than-expected PCR product. In addition, 4 strains were reconstructed by PCR amplifying the deletion cassette from the parent deletion strains using the ‘UP45’ and ‘DOWN45’ primer pairs from the Yeast Gene Deletion Consortium[27], then transforming parent strain BY4741 with the PCR product, selecting for G418-resistant cells, representing homologous recombination replacement of the targeted gene with the kanamycin resistance *KanMX44* cassette (confirmed by PCR as described above). Of these strains, 2 (*bni1ΔKanMX44* and *ubr2ΔKanMX44*) showed growth arrest phenotypes consistent with the corresponding deletion collection strains (ratio of growth $rate_{untreated}/rate_{treated\ with\ alpha\ factor}$ of 1.21 and 1.23, respectively), while 2 strains (*pep7ΔKanMX44* and *vps3ΔKanMX44)* failed to

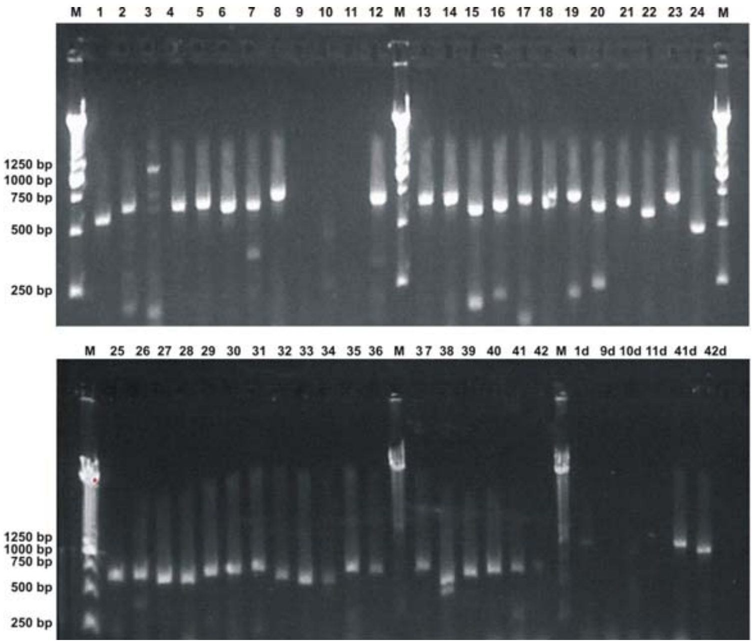

Figure S11: PCR validation of the identity of the deleted gene in 42 deletion strains. 38 of the strains harbor correct deletions; 4 (*dfg10ΔKanMX44*, *suv3ΔKanMX44*, *mdm32ΔKanMX44* and *ydl073wΔKanMX44*) could not be confirmed. Each lane shows results from a PCR assay for the presence of the expected gene deletion and kanamycin insertion cassette. M = marker. *(upper gel)* Lane 1: *yjr050wΔKanMX44*, 2: *yil121wΔKanMX44*, 3: *yor147wΔKanMX44*, 4: *ydr462wΔKanMX4*, 5: *ydl041wΔKanMX4*, 6: *yel004wΔKanMX4*, 7: *ynl264cΔKanMX4*, 8: *yil011wΔKanMX4*, 9: *ydl073wΔKanMX4*, 10: *ypl029wΔKanMX4*, 11: *yil049wΔKanMX4*, 12: *yil084cΔKanMX4* 13: *ylr024cΔKanMX4*, 14: *ydr495cΔKanMX4*, 15: *ydr323cΔKanMX4*, 16: *ynl271cΔKanMX4*, 17: *yil047cΔKanMX4*, 18: *ydl181wΔKanMX4*, 19: *yor036wΔKanMX4,* 20: *yjr152wΔKanMX4*, 21: *ypl161cΔKanMX4*, 22: *yjl062wΔKanMX4*, 23: *ypr023cΔKanMX4*, 24: *yal002wΔKanMX4. (lower gel)* lane 25: *ycl008cΔKanMX4*, 26: *yor089cΔKanMX4*, 27: *ypl002cΔKanMX4*, 28: *ypl065wΔKanMX4*, 29: *ybr078wΔKanMX4*, 30: *ylr417wΔKanMX4*, 31: *yil157cΔKanMX4*, 32: *ydr500cΔKanMX4*, 33: *yhl042wΔKanMX4*, 34: *ygl214wΔKanMX4*, 35: *ygr209cΔKanMX4*, 36: *ynl016wΔKanMX4*, 37: *ykl051wΔKanMX4*, 38: *yal023cΔKanMX4*, 39: *yhl036wΔKanMX4*, 40: *yhr136cΔKanMX4*, 41: *ylr150wΔKanMX4*, 42: *ydr360wΔKanMX4,* 1d: *yjr050wΔKanMX4*, 9d: *ydl073wΔKanMX4*, 10d: *ypl029wΔKanMX4*, 11d: *yil049wΔKanMX4*, 41d: *ylr150wΔKanMX4*, 42d: *ydr360wΔKanMX4*.
Lane labels with the suffix ‘d’ refer to PCR with downstream primer pairs (primers ‘D’ and ‘KanC’).

reproduce the corresponding mating response defective phenotypes (growth rate ratios of 1.84 and 1.78, respectively). Thus, a total of 6 of the complete set of 42 strains assayed could not be validated and were removed from further consideration.

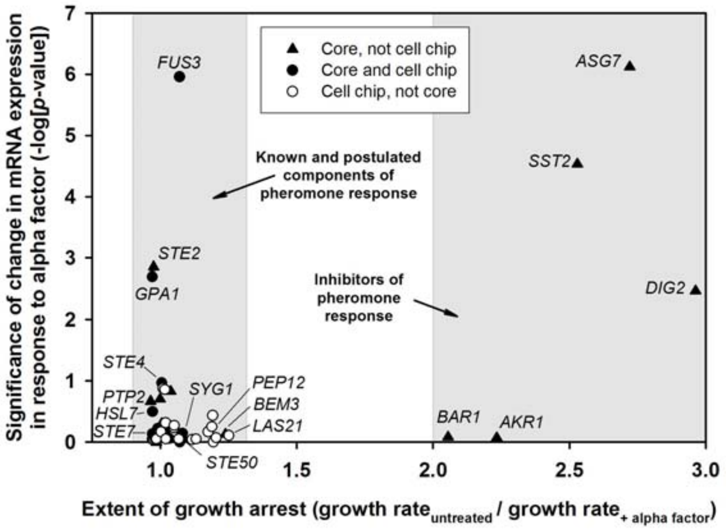

Figure S12: Neither known core pheromone response pathway genes (filled symbols) nor the cell-chip identified components (circles) show a systematic mRNA expression change during alpha factor stimulation. The changes of genes' mRNA levels in wild-type cells in response to addition of 50 nM alpha factor for 30 minutes[8] (y-axis, expressed as the log[probability of a significant expression change]) are plotted opposite the extent of alpha-factor induced growth arrest of strains deleted for the corresponding genes (x-axis; data from this study). Participation in the pathway does not imply changes in mRNA expression of the pathway genes. Instead, core pathway genes fall into two categories (gray regions)—primary pathway components and inhibitors of pathway components; only subsets of each change expression as a function of alpha factor. Other than lacking inhibitors, genes identified in the cell chip assay show the same trend.

<u>Deletion strain response phenotypes correlate poorly with mRNA expression changes</u> We find the extent of alpha factor-induced growth arrest appears largely uncorrelated with the change in expression of the corresponding genes following alpha factor treatment in wild-type cells[8]even for known genes in the core pathway (Figure S12). Instead, the known pathway genes fall into two categories: those whose deletion strains show strong alpha factor-induced growth arrest or those that fail to arrest. The former category is exclusively composed of inhibitors of pheromone response components. Genes in either category may change expression following alpha factor treatment, but the majority of known pathway genes do not, nor do the majority of new genes implicated in the pathway by the combined cell chip/growth inhibition assay. This trend is consistent with signal transduction cascades operating at a post-transcriptional level (i.e., by phosphorylation and protein interactions), not at a transcriptional level. We expect transcriptional changes only in genes downstream of the controlled transcription factors, not upstream, except in those cases where feedback loops are present to modulate the genes' transcription. We can therefore infer that transcriptional feedback control operates for *ASG7*, *SST2*, *FUS3*, and *MFA1* (consistent with the fact that all are known targets of Ste12p[55]), but was not apparent in these experiments for many core pathway genes, such as *FAR1*, *STE5*, *STE7*, *STE11*, *STE50*, *SYG1*, *BEM3*, and *HSL7*.

Visualization of molecules on cell chips We have used cell microarrays to visualize the localization of macromolecules such as DNA and chromosomes by DAPI staining (e.g., see Figure 2) and proteins by antibody staining or other affinity reagents. For example, we have visualized microtubules in cells by probing cell chips with an antibody against tubulin. For this cells were first fixed with formaldehyde, spheroplasted, and then printed as microarrays on to poly-L-lysine coated slides. Cells on the chip were permeabilized in cold methanol for 5 min then in cold acetone. Cell chips were probed with Rat anti-alpha tubulin (YOL1/34; Serotec) as the primary antibody, followed by an FITC conjugated goat anti-rat IgG secondary antibody (Serotec). After washing, the cell chip was additionally probed with DAPI and imaged as before. Figure S13 shows distinct microtubule morphology using the anti-tubulin antibody, demonstrating that it is possible to perform high-throughput detection of proteins in cells on a cell chip by probing with a specific probe.

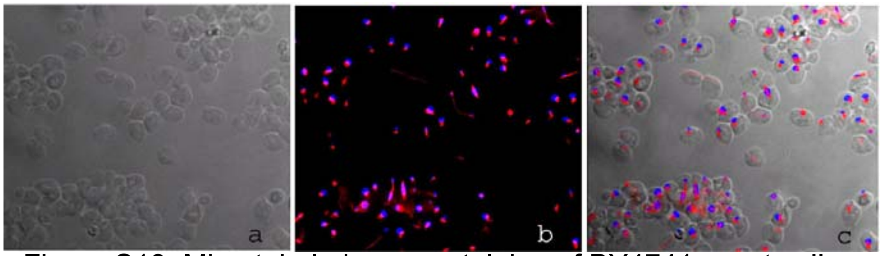

Figure S13: Microtubule immunostaining of BY4741 yeast cells on a cell chip (100X). (a) DIC image (b) DAPI staining (blue) and microtubule staining (red), (c) merged image

REFERENCES

[5] Elion, E.A. Pheromone response, mating and cell biology. Curr Opin Microbiol 3, 573-581 (2000).

[6] Fields, S. Pheromone response in yeast. *Trends Biochem Sci* **15**, 270-273 (1990).

[8] Roberts, C.J. et al. Signaling and circuitry of multiple MAPK pathways revealed by a matrix of global gene expression profiles. *Science* **287**, 873-880 (2000).

[26] Winzeler, E.A. et al. Functional characterization of the S. cerevisiae genome by gene deletion and parallel analysis. *Science* **285**, 901-906 (1999).

[27] Giaever, G. et al. Functional profiling of the Saccharomyces cerevisiae genome. *Nature* **418**, 387-391 (2002).

[31] Robinson, M.D., Grigull, J., Mohammad, N. & Hughes, T.R. FunSpec: a web-based cluster interpreter for yeast. *BMC Bioinformatics* **3**, 35 (2002).

[50] Gad, M., Itoh, A. & Ikai, A. Mapping cell wall polysaccharides of living microbial cells using atomic force microscopy. *Cell Biol Int* **21**, 697-706 (1997).

[52] Burke, D., Dawson, D. & Stearns, T. Methods in Yeast Genetics: A Cold Spring Harbor Laboratory Course Manual. (CSHL Press, Cold Spring Harbor, NY; 2000).

[53] Moore, S.A. Comparison of dose-response curves for alpha factor-induced cell division arrest, agglutination, and projection formation of yeast cells. Implication for the mechanism of alpha factor action. *J Biol Chem* **258**, 13849-13856 (1983).

[55] Zeitlinger, J. et al. Program-specific distribution of a transcription factor dependent on partner transcription factor and MAPK signaling. *Cell* **113**, 395-404 (2003).
